# Vocal education participation and psychological wellbeing among international students in China: mediating roles of emotion regulation and acculturative stress

**DOI:** 10.3389/fpsyg.2026.1822316

**Published:** 2026-05-26

**Authors:** Chen Zejie, Saeed Ahmad, Wang Suyuhan, Abdul Rasool Khoso, Shahnaz Bhutto

**Affiliations:** 1 School of Music and Dance, Northeast Normal University, Changchun, China; 2 Department of Sociology, School of Public Administration, Hohai University, Nanjing, China

**Keywords:** acculturative stress, emotions, international students, music, psychological wellbeing, vocal education

## Abstract

**Introduction:**

International students in China often experience significant psychological challenges during cultural transition. This mixed-methods study investigated whether participation in vocal education programs is associated with psychological wellbeing and examined the statistical mediating roles of emotion regulation and acculturative stress.

**Methods:**

In the quantitative phase, 352 international students from three Nanjing universities completed measures of vocal education participation, psychological wellbeing (WHO-5), emotion regulation (DERS), and acculturative stress (ASSIS). Structural equation modelling was used to test the proposed mediation model. In the qualitative phase, semi-structured interviews were conducted with 22 purposively selected participants.

**Results:**

Vocal education participation was positively associated with psychological wellbeing. Emotion regulation and acculturative stress significantly statistically mediated this association, with indirect effects accounting for a substantial proportion of the total effect; the full model explained a meaningful share of the variance in psychological wellbeing. The final model demonstrated excellent fit. Qualitative themes elucidated three primary mechanisms: emotional expression and release, social connection and belonging, and cultural bridging through music, which enriched the quantitative mediation findings.

**Conclusion:**

Vocal education participation is associated with psychological wellbeing, and this association is statistically mediated by higher levels of emotion regulation and lower levels of acculturative stress. University-based vocal programs may represent accessible, culturally resonant activities that are associated with better mental health among international students in Chinese higher education.

## 1 Introduction

The internationalization of higher education has emerged as a defining feature of twenty-first-century academia, with over 6 million students pursuing education outside their home countries (Antoninis et al., 2023). China is now the third-largest host country for international higher education, attracting around 500,000 international students per year (Gao and De Wit, 2017). Such a large influx presents both challenges and opportunities, particularly regarding students’ psychological adjustment and wellbeing during their stay. International education has significant value for global citizenship; however, the psychological aspects of cultural transition may have far-reaching consequences for students’ mental health and warrant investigation into potential support mechanisms accessible to students and responsive to culture. International students face stressors absent among their domestic counterparts, including language barriers, pedagogical disparities, cultural norm differences, and disengagement from existing support networks (Berry, 1997; Smith and Khawaja, 2011). Acculturative stress or the psychological effects of managing the acculturation process have been consistently found to be negatively associated with mental health outcomes in various international student populations (Sullivan and Kashubeck-West, 2015; Zhang and Goodson, 2011). Chinese-based research has suggested that international students experience greater anxiety, depression, and loneliness due to difficulty in a foreign language, as well as cultural distance (Hang and Zhang, 2023; Jiang et al., 2018; Suyuhan et al., 2026).

Emotion regulation (ER), the ability to assess, evaluate, and adjust emotional reactions, has become an important aspect of psychological adjustment for people travelling abroad in different cultures (Gross, 2015; Song et al., 2025). International students’ emotional intelligence, defined as the capacity for adaptive emotion regulation, positively relates to cultural adaptability and psychological wellbeing (Awasthi et al., 2022; Soufi Amlashi and Forstmeier, 2025). Beyond wellbeing, emotional mechanisms also play a crucial role in academic outcomes; for instance, emotional intelligence has been shown to positively influence academic performance by reducing test anxiety (Vasiou et al., 2025). The relationship between acculturative stress and emotion regulation illustrates the mechanism by which international students’ mental health is crystallised and supported in the future. The emotional regulation patterns explored in this study potentially decrease acculturative stress, indicating that students who more effectively regulate their emotions in the face of cultural challenges may experience lower levels of acculturative stress and improved psychological functioning; thus, emotion regulation interventions can have a considerable impact (Cheung et al., 2020).

Simultaneously, species-appropriate and arts-based approaches have begun to receive much greater interest as accessible, non-stigmatising mental health interventions (Clift and Hancox, 2010; Perkins et al., 2020; Williams et al., 2018). Of these, vocal instruction encompassing formal voice training, singing in choirs, and informal, colloquial song offers a potentially fruitful area of inquiry. A large body of literature shows a positive effect of singing in groups on psychological wellbeing, including the amelioration of symptoms of depression and anxiety, and improved social connectedness across a wide variety of populations (Clift and Hancox, 2010; Dingle et al., 2017; MacRitchie et al., 2020; Williams et al., 2018). Recently, controlled studies found that choir participants among international students showed significantly greater increases in sense of belonging and wellbeing than non-participating controls (Yu et al., 2025). There is a strong association between choir membership and psychosocial resources, such as increased self-esteem, perceived control, meaning and purpose and mood improvement, suggesting that there are several psychological pathways through which members might benefit from vocal engagement (Fryling, 2015).

A few interconnected mechanisms could help to explain the associations between vocal education and psychological wellbeing. From a physiological perspective, singing activates the respiratory and cardiovascular systems, boosts the release of endorphins and oxytocin, and can reduce cortisol levels (Fancourt et al., 2014; Kreutz et al., 2004). Topographically, singing creates spaces in which affect can be organised and modulated: activity is choreographed in time and space, allowing for the expression and regulation of emotions and their engagement at a psychological level (Yao, 2024). On a social basis, and especially in an interconnected environment, singing facilitates interpersonal bonding (Kreutz, 2014; Pearce et al., 2015). Promotes collective identity building and develops network-society support, key assets for international students facing a strange cultural environment (Han et al., 2025). Vocal education participation is associated with international students’ psychological wellbeing through enhanced emotional expression the ability to identify, articulate, and modulate feelings via singing and improved peer communication, which fosters shared identity, social bonding, and acculturative support networks.

### 1.1 Theoretical framework

#### 1.1.1 Emotion regulation framework

The extended process model of emotion regulation suggests that engagement with vocal education is associated with improvements in regulatory capacities (Gross, 2015). Singing involves attending to emotional content, appraising musical and lyrical material cognitively, and modulating expressive response processes that mirror key aspects of adaptive emotion regulation. Indeed, singing activities are necessarily emotionally aware, expressive, and management-based (Fancourt et al., 2014). Engaging in them regularly *may be associated with* the development of these skills. In the acculturative stress framework, acculturative stress is conceptualised as a central construct underlying cross-cultural adaptation. Engaging in activities that support cultural learning, opportunities for positive intergroup contact with the new culture, and belonging to the host society may be related to lower stress regarding their own cultural transition (Berry, 1997). Programs such as those in parts of New York City, where vocal education is based on Chinese musical traditions or on interactions with Chinese peers, may fulfil these functions. The social identity approach to health (Jetten et al., 2017), indicates that psychological resources for mental health are derived from group memberships. Vocal education grouping to find out about university life helps international students develop a significant social identity when creating bonds with others who have similar interests, whilst being given a sense of cohesion throughout the wider bubble of the student community on campus.

### 1.2 The present study

Despite theoretical support, empirical studies on vocal education and international students’ wellbeing are scarce. This study fills the gap using a sequential mixed-methods design at three universities in Nanjing, China. Our research questions were:

*RQ1*: Is vocal education participation associated with psychological wellbeing among international students in China?

*RQ2*: Do emotion regulation and acculturative stress mediate this relationship?

*RQ3*: How do international students experience vocal education participation, and what mechanisms do they perceive as influencing their wellbeing?

We hypothesised:

*H1*: Vocal education participation would be positively associated with psychological wellbeing.

*H2*: Emotion regulation would statistically mediate the association between vocal education participation and psychological wellbeing.

*H3*: Acculturative stress would statistically mediate the association between vocal education participation and psychological wellbeing.

*H4*: Emotion regulation and acculturative stress would function as sequential statistical mediators of the association between vocal education participation and psychological wellbeing.

## 2 Methods

### 2.1 Study design

This study used a sequential explanatory mixed-methods design (Creswell and Clark, 2007) with two linked phases. The quantitative phase tested hypothesised relationships among vocal education participation, emotion regulation, acculturative stress, and psychological wellbeing, while the qualitative phase employed semi-structured interviews with a purposively selected subset to explore participants’ experiences and clarify the mechanisms underlying the quantitative findings.

### 2.2 Participants

#### 2.2.1 Quantitative sample

The participants were recruited from three universities in Nanjing, China: Hohai University, Nanjing University and Southeast University. Inclusion criteria were: (1) active international student status (holding a non-Chinese passport and student visa), (2) ≥ 18 years of age, (3) enrollment in a degree-seeking or formal exchange program, and (4) duration of stay in China ≥ 3 months.

Using the Satorra Saris method for power analysis in structural equation modeling (Satorra and Saris, 1985) a sample size of 300 participants was determined to be sufficient to detect small-to-medium misspecification effects (RMSEA ≥ 0.05) with a power of 0.80, given the complexity of the hypothesized measurement and structural models (4 latent variables, 20 observed indicators, *α* = 0.05). Furthermore, the sample satisfies the commonly recommended *N*:*q* rule (Jackson, 2003), yielding a ratio of 17.6:1 (352 participants to 20 indicators), which exceeds the minimum threshold of 10:1. Consequently, the final sample of 352 participants provides adequate statistical power for the proposed SEM analyses.

#### 2.2.2 Qualitative sample

For an in-depth interview, we purposively selected 22 individuals for qualitative interviews. To maximize diversity, the selection criteria considered different levels of vocal participation (11 regular, 7 occasional, 4 non-participants), varying universities and countries of origin, length of residence in China, and other demographic characteristics. The qualitative sample included 12 women and 10 men from 14 countries, with a mean age of 25.1 years.

### 2.3 Procedure

Data collection occurred between September 2025 and January 2026. In the quantitative phase (September 2025–December 2025), participants completed the survey either online via Qualtrics or on paper. The survey required approximately 25–30 min. All participants provided informed consent, and participation was voluntary. In the qualitative phase (January–February 2026), semi-structured interviews were conducted in private rooms, lasted 45–90 min, and were audio-recorded with permission. Participation was voluntary. Interviews continued until thematic saturation (Guest et al., 2006). The study protocol was approved by the Institutional Review Board of Hohai University (Protocol #2024-089). Permission to recruit participants was obtained from the relevant academic departments at Nanjing University and Southeast University.

### 2.4 Measures

All instruments were administered in English, the primary language of instruction for most international programs and the common language among the diverse international student population. A composite measure of student vocal education participation was developed to contextualize it within typical higher-education settings in China. Considering participation not as a “reflection” of an underlying latent variable, but rather as a formative construct (before the analysis began), (i.e., type, frequency, and duration are all indicators that account for participation). The instrument evaluated three dimensions: (1) frequency (never, occasional, monthly, weekly, multiple times weekly), (2) type of singing experience (formal voice lessons, university choir, informal singing groups, individual practice, religious singing, other), and (3) duration (2 years). Vocal education participation was specified as a formative (rather than reflective) construct because participation comprises distinct, non-interchangeable dimensions (frequency, type diversity, and duration) that jointly define the construct rather than reflecting an underlying latent trait. In a reflective model, changes in the latent construct would be expected to cause changes in all indicators (e.g., more ‘participation’ would increase both frequency and duration). However, a student could attend frequently (high frequency) but only sing informally (low type diversity), or participate for many years (long duration) but only occasionally (low frequency). These dimensions are not necessarily correlated, making a formative specification more appropriate (Diamantopoulos and Siguaw, 2006). Because vocal education participation was specified as a formative construct, measurement quality was assessed through the significance and magnitude of indicator weights and by testing for multicollinearity, rather than by internal consistency metrics such as Cronbach’s *α*. Indicator weights ranged from 0.31 to 0.48 (all *p* < 0.001), and variance inflation factors were below 2.5, indicating no substantial multicollinearity. Psychological wellbeing was assessed with the World Health Organization Five WellBeing Index (Fung et al., 2022; Topp et al., 2015), a 5-item index in which respondents scored statements such as “I have felt cheerful and in good spirits” on a 6-point scale from 0 (at no time) to 5 (all of the time) over the past 2 weeks. Scores can range from 0 to 25, with higher scores indicating better wellbeing. The WHO-5 is cross-culturally validated (Fung et al., 2022), and the scale showed excellent reliability in this study (*α* = 0.89). This questionnaire included the Difficulties in Emotion Regulation Scale (DERS; Gratz and Roemer, 2004). which comprises 36 items on a five-point scale (from 1 = rarely to 5 = almost always) measuring six aspects of difficulty controlling emotions. Higher scores indicate greater difficulty (worse emotion regulation); however, to improve interpretability in mediation analyses, scores were reversed so that higher scores indicate better emotion regulation. The DERS has shown robust psychometric properties in cross-cultural studies (Soufi Amlashi and Forstmeier, 2025) and had high internal consistency in this sample as well (*α* = 0.92). Acculturative stress was measured with the Acculturative Stress Scale for International Students (ASSIS; Sandhu and Asrabadi, 1994), a 36-item scale with seven subscales, each item rated on a 5-point Likert-type scale ranging from 1 (strongly disagree) to 5 (strongly agree). Total scores on the measure range from 36 to 180, with higher scores representing greater acculturative stress. The ASSIS is often employed in international studies with students, such as those conducted in China (Hang and Zhang, 2023; Jiang et al., 2018; Suyuhan et al., 2026), and showed excellent reliability here (α = 0.93). Participants also reported demographic and control variables (age, Sex, country of origin, duration of residence in China, self-rated Chinese language ability (1–5), academic level, and previous musical experience).

### 2.5 Quantitative data analysis

Quantitative data were analysed in a sequential procedure using three software packages. Preliminary descriptive statistics, bivariate correlations, and demographic comparisons were computed in SPSS version 28. Confirmatory factor analysis (CFA) and alternative model comparisons were performed in Mplus 8.10 with full information maximum likelihood (FIML) estimation to handle missing data. The hypothesised structural equation model (SEM) and bootstrapped mediation tests (5,000 resamples) were estimated using the semopy library (version 2.5) in Python, chosen for its flexible specification of complex path models. This integrated approach ensured that each analytical stage was conducted with the most appropriate tool while maintaining methodological coherence.

*Common method bias*: To assess the potential for common method bias, Harman’s single-factor test was conducted. All measured indicators were loaded into an exploratory factor analysis with an unrotated solution. The first factor accounted for 28.7% of the total variance, which is below the recommended 50% threshold (Podsakoff et al., 2012). While this result suggests that common method variance is unlikely to substantially confound the interpretation of the findings, it is important to acknowledge the limitations of this approach. The single-factor test is a widely used but relatively insensitive *post hoc* diagnostic; it does not statistically control for method effects and may fail to detect bias when complex measurement models are involved (Podsakoff et al., 2012; Tehseen et al., 2017) Consequently, the results of this test should be interpreted with appropriate caution.

*Alternative model testing*: We tested two alternative models: Model A (Reverse Causation): Psychological wellbeing → Emotion Regulation → Vocal Education Participation; and Model B (Alternative Mediation): Vocal Education Participation → Acculturative Stress → Emotion Regulation → Psychological Wellbeing. Model comparisons used Akaike Information Criterion (AIC) and Bayesian Information Criterion (BIC). Missing data were minimal (<3%) and handled using full information maximum likelihood (FIML) estimation in Mplus (Enders, 2010).

### 2.6 Qualitative data analysis

Interview recordings were transcribed verbatim and imported into NVivo 14. Data analysis followed (Braun and Clarke, 2021) six-phase approach to reflexive thematic analysis (RTA), selected for its theoretical flexibility and emphasis on researcher reflexivity.

The analysis proceeded iteratively. Phase 1 (familiarisation) began during transcription and continued through repeated reading of all 22 transcripts; the first author (C.Z.) recorded analytic memos documenting initial impressions and emerging patterns. Phase 2 (systematic coding) involved generating semantic codes (e.g., “singing releases tension”) alongside latent codes capturing underlying assumptions (e.g., “emotional naming as coping”). A second researcher (S.A.) independently coded four transcripts (18% of the sample); discrepancies were resolved through discussion, refining the coding framework. Phase 3 (generating initial themes) employed thematic mapping to cluster codes into candidate themes. Phase 4 (reviewing and developing themes) assessed themes for internal coherence and external distinctiveness. Candidate themes were merged (e.g., “shared identity” and “practical support” into “social connection and belonging”), subsumed, or discarded when insufficiently prevalent. Phase 5 (defining and naming themes) involved refining thematic definitions and selecting representative extracts. Phase 6 (producing the report) integrated themes with quantitative findings.

The analysis was mostly inductive: initial coding was data-driven, although theoretical sensitivity to concepts emotion regulation stages (Gross, 2015), acculturation and stress buffers (Berry, 1997), and social identity mechanisms (Jetten et al., 2017) informed later interpretive stages, consistent with reflexive thematic analysis principles (Braun and Clarke, 2021).

#### 2.6.1 Reflexivity and researcher positionality

The research team comprised backgrounds in music education (C.Z.), sociology (S.A., W.S., A.R.K., S.B.), and public health. Regular team meetings served as a reflexive space for surfacing interpretive assumptions; analytic memos documented how disciplinary backgrounds and personal experiences (including several members’ own international student experiences) shaped coding and interpretation.

#### 2.6.2 Language, translation, and interview protocol

Interviews were conducted in English, the common language among participants and the medium of instruction for most academic programs; no translation was required. The semi-structured protocol, developed after preliminary quantitative analysis, explored experiences with vocal education and potential mechanisms underlying quantitative associations. Core questions addressed participation experiences, emotional connections, social relationships, and influences on cross-cultural adaptation. The protocol was piloted with two international students not included in the final sample, with minor wording adjustments made thereafter.

#### 2.6.3 Thematic saturation

Saturation was approached pragmatically (Braun and Clarke, 2021; Guest et al., 2006). Interviews were analysed sequentially after each batch of three to four interviews. Saturation was operationally defined as the point at which no new codes emerged and existing themes could adequately accommodate new data without substantial revision. This was reached after 18 interviews. The final four interviews confirmed the existing thematic structure (no new codes, and all new data fit within the three established themes and subthemes). The sample of 22 aligns with recommendations for moderate-to-high interpretive richness in RTA (Braun and Clarke, 2021).

### 2.7 Ethical considerations and informed consent

The study protocol was approved by the Ethics Review Committee of the School of Public Administration, Hohai University (Reference: 20251103). All semi-structured interviews were conducted by C.Z. (doctoral candidate) and S.A. (doctoral candidate). Neither interviewer held any teaching, supervisory, evaluative, or administrative authority over participants; specifically, C.Z. had no prior relationship with any participant, and S.A. had no role in academic assessment, visa status, or vocal program oversight. No interviews were conducted by choir directors, voice instructors, or faculty from participants’ home departments. To prevent coercion, recruitment used general university mailing lists and public notice boards (not classes, choir rehearsals, or advising meetings). The informed consent form and oral preamble explicitly stated: “The interviewers are not your teachers, advisors, or choir directors. Your participation or non-participation will not affect your grades, vocal program standing, or student visa.” Interviews were conducted in neutral library study rooms, and participants were reminded before each interview that they could skip any question or withdraw without consequence.

## 3 Results

### 3.1 Participant characteristics

Table 1 presents the demographic profile of the respondents, international students from Hohai University, Nanjing University, and Southeast University. Participants across diverse regions of Asia (47.7%) and Africa (52.3%), aged 24.9 years on average (SD = 4.4), and slightly more female than male (53.7%). The most extensive subgroup was that of Master’s students (*n* = 177, 50.3%). Examination of vocal education attendance revealed that 27.6% attended a choir or singing group/lessons regularly, 32.1% attended occasionally, and 40.3% had no participation, indicating strong variability in the focal independent variable.

**Table 1:** Demographic characteristics of quantitative sample (*N* = 352).

Characteristic	Category	*n*	%
Sex	Female	189	53.7
	Male	159	45.2
	Non-binary/prefer not to say	4	1.1
Age group	18–22 years	112	31.8
	23–27 years	169	48.0
	28–32 years	52	14.8
	33 + years	19	5.4
Region of origin	Asia (excluding China)	168	47.7
	Africa	184	52.3
University	Hohai University	118	33.5
	Nanjing University	124	35.2
	Southeast University	110	31.3
Academic level	Undergraduate	114	32.4
	Master’s	177	50.3
	Doctoral	61	17.3
Vocal participation	Regular (weekly+)	97	27.6
	Occasional (monthly)	113	32.1
	None	142	40.3

#### 3.1.1 Comparison of participation groups

One-way ANOVAs and chi-square tests were conducted to compare regular (*n* = 97), occasional (*n* = 113), and non-participants (*n* = 142). No significant group differences were observed for age, *F*(2, 349) = 0.87, *p* = 0.42; Sex, *χ*^2^(4) = 3.21, *p* = 0.52; region of origin, *χ*^2^(2) = 1.94, *p* = 0.38; or length of residence in China, *F*(2, 349) = 1.52, *p* = 0.22. A significant difference was found in self-rated Chinese language proficiency, with regular participants reporting higher scores (*M* = 3.67, *SD* = 0.89) than non-participants (*M* = 3.12, *SD* = 0.94; *p* = 0.008). Accordingly, subsequent analyses included self-rated Chinese language proficiency as a control variable.

### 3.2 Preliminary analyses

**Figure 1:**
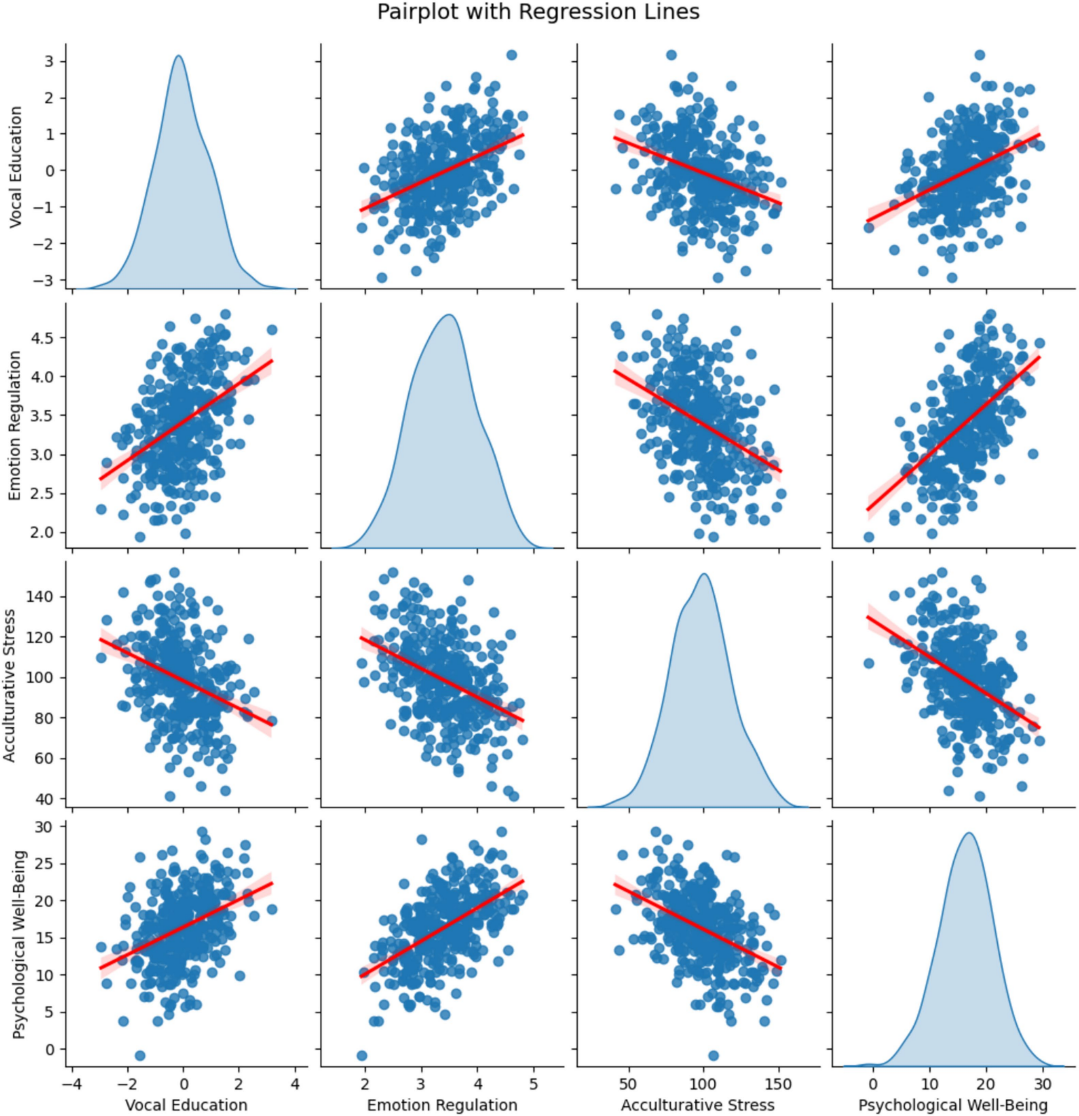
Descriptive statistics and bivariate correlations among study variables (*N* = 352).

Figure 1 shows descriptive statistics and bivariate correlations for all study variables. Vocal education participation was associated with psychological wellbeing (*r* = 0.40, *p* < 0.001) and emotion regulation (*r* = 0.43, *p* <0.001), and was inversely associated with acculturative stress (*r* = −0.38, *p* < 0.001). There was a positive correlation between emotion regulation and wellbeing (*r* = 0.54, *p* < 0.001), and acculturative stress was negatively related to wellbeing (*r* = −0.51, *p* < 0.001). Analyses of demographic variables showed that length of residence was significantly positively associated with wellbeing (*r* = 0.20, *p* < 0.01) and negatively correlated with acculturative stress (*r* = −0.23, *p* < 0.001). The same pattern was found for Chinese language proficiency. Emotion regulation was significantly higher in women than men (*t* = 2.38, *p* < 0.05). These factors were used as covariates in the analysis that followed.

### 3.3 Measurement model

Confirmatory factor analysis (CFA) was conducted to assess the measurement model prior to testing structural relationships. The model comprised three latent constructs: emotion regulation (with six subscales as indicators), acculturative stress (with seven subscales as indicators), and psychological wellbeing (with five items as indicators) and one formative construct (vocal education participation, with three indicators: frequency, type diversity, and duration). For the formative construct, indicator weights ranged from 0.31 to 0.48 (all *p* < 0.001), and variance inflation factors (VIFs) were < 2.5, indicating no significant multicollinearity.

The initial measurement model demonstrated acceptable fit (*χ*^2^/df = 2.31, CFI = 0.94, TLI = 0.93, RMSEA = 0.06, SRMR = 0.05). After correlating residuals between two emotion regulation subscales (awareness and clarity) based on conceptual overlap and modification indices, model fit improved substantially (*χ*^2^/df = 2.04, CFI = 0.97, TLI = 0.96, RMSEA = 0.04, SRMR = 0.04). All factor loadings for reflective constructs were significant (*p* < 0.001) and exceeded 0.60, indicating adequate measurement.

### 3.4 Common method bias

Harman’s single-factor test was conducted to assess potential common method bias. All observed indicators were loaded into an exploratory factor analysis with an unrotated solution. The first factor accounted for 28.7% of total variance, substantially below the 50% threshold, suggesting that common method variance is unlikely to confound interpretation of the findings.

### 3.5 Hypothesis testing

#### 3.5.1 Direct association (H1)

The first analysis examined the direct association between participation in vocal education and psychological wellbeing, without including mediators. This model demonstrated good fit (*χ*^2^/df = 2.08, CFI = 0.98, TLI = 0.97, RMSEA = 0.04, SRMR = 0.03). Vocal education participation was positively associated with psychological wellbeing (*β* = 0.36, 95% CI [0.24, 0.48], *p* < 0.001), supporting H1.

#### 3.5.2 Primary analysis: parallel mediation (H2 and H3)

**Figure 2:**
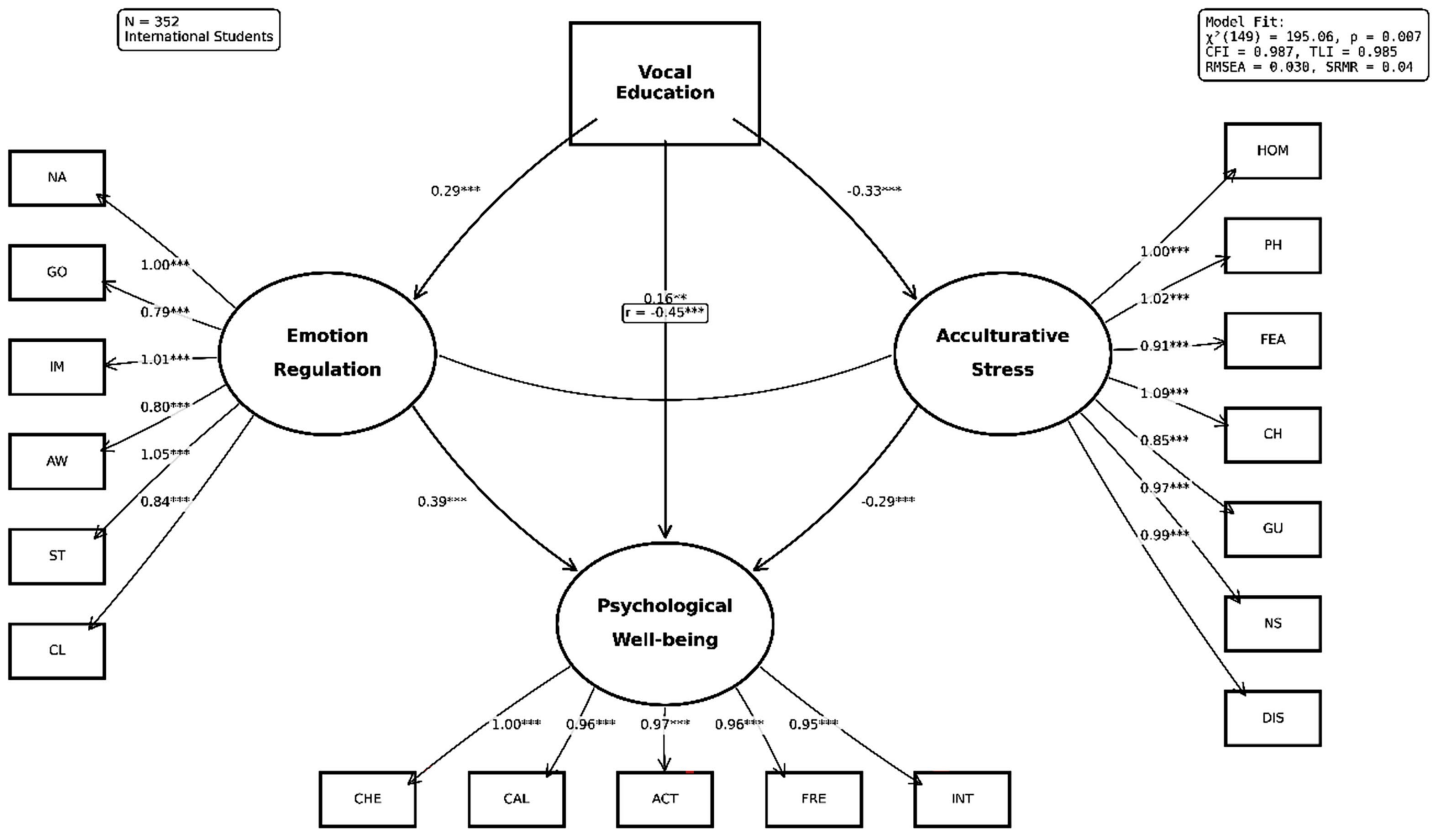
Structural equation model of parallel mediation effects.

The primary analysis tested a parallel mediation model in which emotion regulation and acculturative stress were specified as simultaneous mediators of the association between participation in vocal education and psychological wellbeing (Figure 2). This model demonstrated excellent fit to the data (*χ*^2^(149) = 195.06, *p* = 0.007, CFI = 0.987, TLI = 0.985, RMSEA = 0.030, SRMR = 0.04).

As shown in Table 2 and Figure 2, vocal education participation was positively associated with emotion regulation (*β* = 0.45, 95% CI [0.35, 0.55], *p* < 0.001) and negatively associated with acculturative stress (*β* = −0.40, 95% CI [−0.52, −0.28], *p* < 0.001). In turn, emotion regulation showed a positive association with psychological wellbeing (*β* = 0.37, 95% CI [0.27, 0.47], *p* < 0.001), while acculturative stress showed a negative association with psychological wellbeing (*β* = −0.33, 95% CI [−0.45, −0.21], *p* < 0.001). After representing these mediators, the direct association between participation in vocal education and psychological wellbeing remained significant (*β* = 0.15, 95% CI [0.05, 0.25], *p* = 0.007), indicating partial statistical mediation.

**Table 2:** Direct and indirect effects in the parallel mediation model.

Path	*β*	SE	95% CI	*p*
Direct effects				
Vocal education → wellbeing (total)	0.45	0.06	[0.33, 0.57]	<0.001
Vocal education → wellbeing (direct)	0.15	0.05	[0.05, 0.25]	0.007
Vocal education → emotion regulation	0.45	0.05	[0.35, 0.55]	<0.001
Vocal education → acculturative stress	−0.40	0.06	[−0.52, −0.28]	<0.001
Emotion regulation → wellbeing	0.37	0.05	[0.27, 0.47]	<0.001
Acculturative stress → wellbeing	−0.33	0.06	[−0.45, −0.21]	<0.001
Indirect effects				
Via emotion regulation	0.17	0.03	[0.12, 0.23]	<0.001
Via acculturative stress	0.13	0.03	[0.08, 0.19]	<0.001
Total indirect	0.30	0.04	[0.22, 0.38]	<0.001

Bootstrap confidence intervals based on 5,000 resamples.

A bootstrap analysis with 5,000 resamples was conducted to assess the statistical significance of the indirect effects. The indirect effect through emotion regulation was 0.17 (95% CI [0.12, 0.23]), supporting H2. The indirect effect through acculturative stress was 0.13 (95% CI [0.08, 0.19]), supporting H3. The total indirect effect (sum of both parallel mediators) was 0.30 (95% CI [0.22, 0.38]), accounting for 61.8% of the total association between vocal education participation and psychological wellbeing (total effect *β* = 0.45). The model explained 44% of the variance in psychological wellbeing.

#### 3.5.3 Secondary analysis: exploratory sequential mediation (H4)

As a secondary, exploratory analysis, we also examined a sequential mediation model that proposed the pathway from vocal education participation through emotion regulation and acculturative stress to psychological wellbeing. This model was driven by theoretical considerations proposing that advanced emotion regulation skills could enable students to tackle better acculturative challenges, which, in turn, would be associated with increased psychological wellbeing.

The sequential mediation model provided an acceptable fit (*χ*^2^/df = 2.28, CFI = 0.96, TLI = 0.95, RMSEA = 0.05, SRMR = 0.05). The sequential indirect effect (vocal education → emotion regulation → acculturative stress → wellbeing) was significant but small in magnitude, *β* = 0.07, 95% CI [0.04, 0.11], *p* < 0.001, offering initial confirmation for H4. However, this sequential pathway contributed to a much smaller share of the total association (15.6%) compared with the parallel mediation pathways in the primary analysis. As the data are cross-sectional, this sequential model should be viewed as exploratory rather than confirmatory; however, it does highlight key variables of interest in a novel way that will guide subsequent research.

#### 3.5.4 Alternative model testing

To address concerns about directionality inherent in cross-sectional mediation analyses, we tested two alternative model specifications. These models do not establish causality but provide information about which directional specification is more consistent with the observed data.

- *Model A (Reverse Causation)*: Psychological wellbeing → Emotion Regulation → Vocal Education Participation
- *Model B (Alternative Mediation):* Vocal Education Participation → Acculturative Stress → Emotion Regulation → Psychological Wellbeing

**Figure 3:**
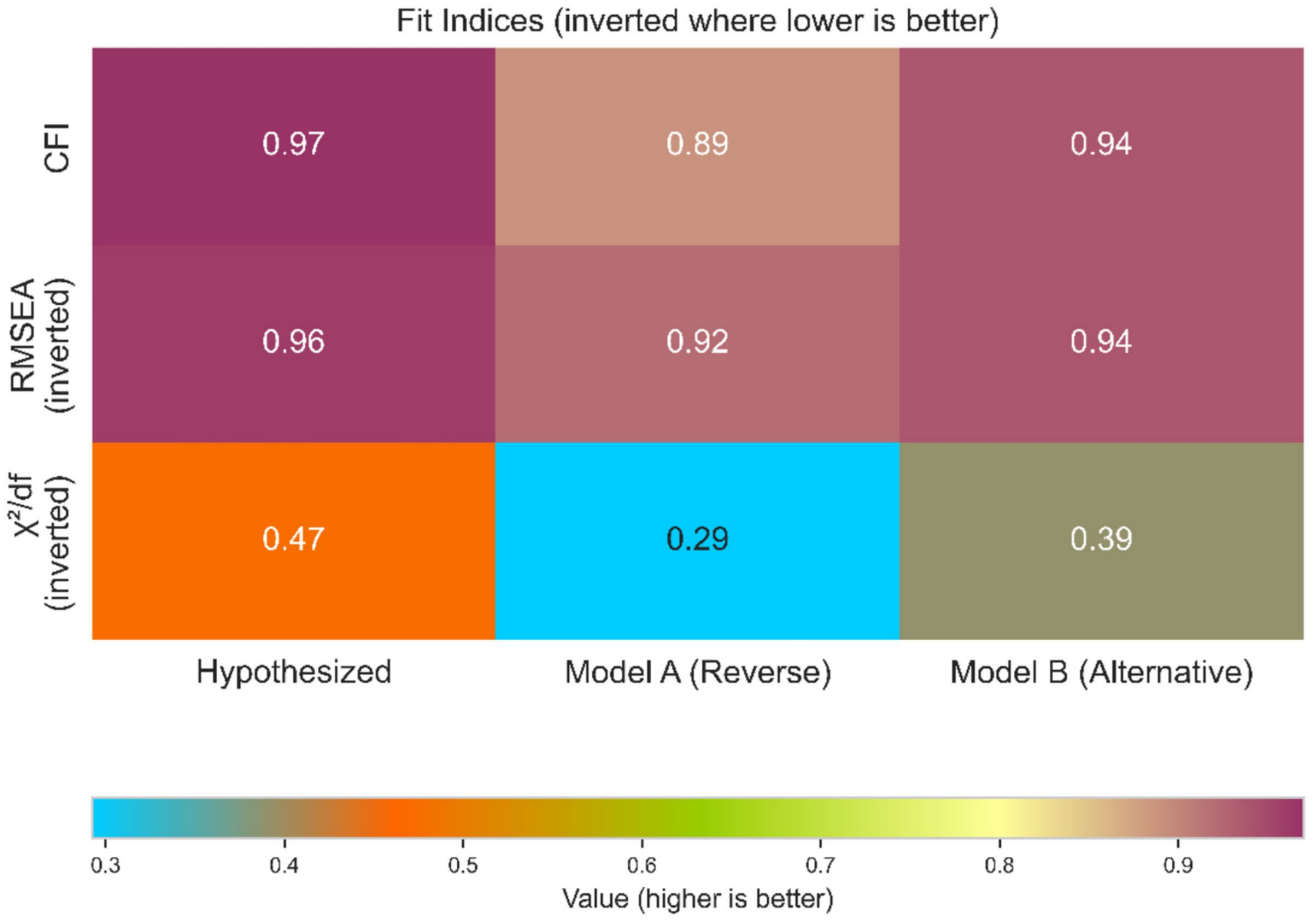
Model comparison statistics for alternative directional specifications.

Figure 3 presents model comparison statistics evaluating alternative directional specifications. The hypothesised parallel mediation model demonstrated superior fit across all indices (*χ*^2^(149) = 195.06, *p* = 0.007, CFI = 0.987, TLI = 0.985, RMSEA = 0.030, SRMR = 0.04) compared to the reverse causation model (Model A: *χ*^2^/df = 3.42, CFI = 0.89, RMSEA = 0.08) and the alternative sequential model (Model B: *χ*^2^/df = 2.56, CFI = 0.94, RMSEA = 0.06). The lower AIC and BIC values for the hypothesised model (12,845 and 13,102, respectively) indicate that this specification offers the most parsimonious account of the relationships among the study variables, including empirical support for our proposed directional sequence flowing from vocal education participation through the mediator’s pathway to psychological wellbeing.

### 3.6 Qualitative findings

#### 3.6.1 Reflexive thematic analysis

Reflexive thematic analysis of the 22 semi-structured interviews generated three overarching themes, each comprising two to three subthemes, that illuminate the experiential mechanisms linking vocal education participation to psychological wellbeing among international students in China. These themes Emotional Expression and Release, Social Connection and Belonging, and Cultural Bridging Through Music represent patterns of shared meaning across the dataset. Each theme is presented below, accompanied by supporting data extracts and analysis of thematic nuance, inter-theme relationships, and variation across participants.

#### 3.6.2 Theme 1: emotional expression and release

This theme captures participants’ accounts of singing as a vehicle for emotional processing, encompassing both spontaneous cathartic release and more structured, reflective forms of emotional engagement. The theme comprised two distinct but interrelated subthemes: *cathartic release* and *structured emotional processing*.

##### 3.6.2.1 Subtheme 1.1: cathartic release

The majority of participants (18 of 22) described singing as a means of discharging accumulated emotional tension, particularly tension arising from acculturative stressors. This release was often described in physiological terms, emphasising the embodied nature of emotional relief. A 26-year-old female participant from Pakistan, a regular choir member, captured this experience:

“*When I miss my home or feel frustrated with language, singing really helps me get it out. It’s not about singing well sometimes I’m crying while we sing. After choir practice, I always feel lighter, like I have emptied some heavy baggage. My shoulders drop. I breathe deeper. I didn’t expect music to affect my body like that.*” (P08, female, 26, Pakistan, regular choir).

A 31-year-old male participant from Nigeria, who had been taking individual voice lessons for 8 months, similarly emphasised the physical dimension of release:

“*Singing is physical. Every day was so tense during the first couple of months in Nanjing. My teacher would just keep saying, ‘Relax, breathe from your diaphragm.’ At first, I thought it was just technique. But I realised it was about teaching my body to let go of stress. When I’m singing, I can feel the tension leaving my neck, my jaw. I carry that into my daily life now.*” (P14, male, 31, Nigeria, regular voice lessons).

For several participants, this cathartic function was most pronounced during periods of acute stress. A 29-year-old male participant from Nepal, who attended choir sporadically, described turning to singing during a period of research-related distress:

“*There was a month when my supervisor was very critical, and I felt like I was failing. I stopped going to choir for a few weeks. When I finally went back, we sang this powerful piece very loud, very emotional and I broke down during rehearsal. The conductor just let me stand there and sing through it. Afterward, I felt like something had been released that I didn’t even know I was holding. It wasn’t like talking to a counsellor. It was different. More direct.*” (P05, male, 29, Nepal, sporadic choir).

Notably, even participants who had discontinued vocal education involvement referenced the loss of this cathartic outlet. A 23-year-old female participant from Bangladesh, who had left the university choir due to academic pressures, reflected:

“*I didn’t realise how much I needed it until I stopped. Now when I’m stressed, I just sit with it. I don’t have that release anymore. Sometimes I sing by myself in my room, but it’s not the same. The group practice had a structure to it that forced me to let go for two hours.*” (P19, female, 23, Bangladesh, former regular).

##### 3.6.2.2 Subtheme 1.2: structured emotional processing

Beyond immediate catharsis, participants described how vocal education facilitated a more reflective engagement with emotion. This subtheme captured processes of emotional identification, labelling, and meaning-making that participants explicitly linked to improved emotional self-awareness. A 22-year-old female participant from Algeria, an occasional choir member, articulated this process:

“*When we start with a new piece, we discuss what the emotional content is: joyful, sad, longing? You have to understand to sing it well. So, I began to reflect more clearly on my own feelings. Before, I suffered or was depressed without knowing why. Now I can recognise, ‘This is loneliness. This is frustration at being unable to understand.’ Naming it is the way I cope with it.*” (P03, female, 22, Algeria, occasional choir).

A 24-year-old male participant from Egypt, who had been taking voice lessons for over a year, described how his teacher’s pedagogical approach modelled emotional reflection in ways that transferred to daily life:

“*My voice teacher often asks, ‘What emotion are you trying to convey?’ before we work on technique. It seemed strange to me at first I just wanted to sing the notes correctly. But I understand now that once I know the emotion, it makes sense in the sound I have to make. I have started to do a small quantity of the same thing in my own life, asking myself what I am feeling before I go into problem-solving mode. It helps me not react so quickly when I am frustrated with Chinese bureaucracy or language misunderstandings.*” (P12, male, 24, Egypt, regular voice lessons).

The relationship between these two subthemes cathartic release and structured processing was not linear but rather recursive. Participants described how immediate release sometimes enabled subsequent reflection, while structured emotional work deepened the cathartic experience over time. A 27-year-old female participant from Indonesia, a regular choir member, explained:

“*At first, singing was just about letting everything out. I would cry during rehearsal and not really know why. But over time, I started to understand the emotions in the music more deeply. Now I can use singing to process things deliberately. The release still happens, but it’s not just an explosion anymore. It’s more like I’m learning to direct the emotion, to shape it. That feels more sustainable.*” (P11, female, 27, Indonesia, regular choir).

#### 3.6.3 Theme 2: social connection and belonging

This theme encompassed participants’ accounts of vocal groups as sites of relational connection that countered the social isolation commonly associated with international student experience. Two subthemes captured distinct but overlapping dimensions: *shared identity* and *supportive community*.

##### 3.6.3.1 Subtheme 2.1: shared identity

Participants consistently described vocal groups as spaces where national, linguistic, and cultural differences receded, replaced by a collective identity as singers. This shared identity was often described as providing relief from the hypervisibility experienced as international students in academic contexts. A 27-year-old female participant from Indonesia expressed this with particular clarity:

“*In choir, we’re not foreigners or locals, we’re just singers. Sharing challenges and successes creates deep bonds, and for those two hours, I truly belong. There’s no question of whose Chinese is better or who is from where. We are all just trying to make the piece work together. That feeling that I am not being evaluated as a foreigner is so precious to me.*” (P11, female, 27, Indonesia, regular choir).

A 24-year-old female participant from Sri Lanka, who attended choir weekly, described how this shared identity contrasted with her experiences elsewhere on campus:

“*I am usually the only foreign student in my classes. I feel strange; I worry about my Chinese, which makes me afraid to talk. In class, I am always aware of being different. However, in the university chorus, many international and Chinese students welcome us. It is the one place on campus where I have absolutely no memory of being a foreigner. We are just singers. The Chinese students don’t treat me differently; they just hand me the music and say, ‘Let’s work on this section.’ That normalcy is everything.*” (P06, female, 24, Sri Lanka, frequent choir).

The construction of shared identity was not automatic but actively cultivated, particularly by choir conductors and group leaders. A 28-year-old male participant from Morocco described how his choir conductor deliberately fostered an inclusive ethos:

“*Our conductor always says, ‘There is no international choir or Chinese choir. There is only our choir.’ She translates lyrics into English so everyone understands. She chooses music from different countries, not just Chinese repertoire. I think she understands that the choir is not just about music for some of us. It is about creating a place where we all belong together. She makes that explicit, and it matters.*” (P16, male, 28, Morocco, regular choir).

##### 3.6.3.2 Subtheme 2.2: supportive community

Beyond identity transformation, vocal groups provided tangible and emotional support that directly alleviated acculturative stress. This subtheme captured participants’ accounts of practical assistance, emotional validation, and the formation of meaningful relationships that extended beyond rehearsal spaces. A 29-year-old male participant from Nepal described how choir members became a surrogate family:

“*The older choir members became like big siblings. When I arrived, I did not know how to open a bank account, register with the police, or use the delivery apps. The choir members did everything for me. More importantly, when I was feeling down about my research or missed home, they listened. That support system made me feel at home here faster than anything else. I would tell any new international student: join something, anything, that meets regularly. The relationships form naturally when you are making music together.*” (P05, male, 29, Nepal, sporadic choir).

A 23-year-old female participant from Bangladesh highlighted the role of digital communication in sustaining this community:

“*We have a WeChat group for our choir, and it is very active. People share information about concerts, but also information about life: where to find ingredients from home, how to handle visa extensions, which doctors speak English. If I am stressed about something, I can always ask them for help, and someone will step up. It takes so much pressure off. I don’t have to figure everything out alone anymore. Just knowing that there are people who will help if I need it makes daily life less exhausting.*” (P19, female, 23, Bangladesh, regular choir).

Importantly, this supportive function was not uniformly experienced. Four participants who attended vocal groups irregularly described feeling less embedded in these support networks, suggesting that sustained participation may be necessary to realise the full protective benefits. A 25-year-old male participant from Kenya, who had attended only three choir rehearsals over 6 months, reflected:

“*I went a few times, but I always felt like an outsider. Everyone seemed to already know each other. No one was unfriendly, but I didn’t know how to break into the group. I stopped going because I felt more lonely there than I did by myself. Maybe if I had kept going, it would have changed. But I didn’t have the energy to push through that awkward phase.*” (P21, male, 25, Kenya, minimal participation).

This counterexample underscores that the community-building function of vocal groups may require intentional facilitation, particularly to integrate newcomers and those who may be more socially hesitant.

#### 3.6.4 Theme 3: cultural bridging through music

This theme captured participants’ accounts of vocal education as a site of cultural learning and identity negotiation. Three subthemes emerged: *language acquisition through song*, *understanding cultural values*, and *cultural hybridity and meaning-making*.

##### 3.6.4.1 Subtheme 3.1: language acquisition through Song

For participants who sang Chinese repertoire, the process of learning lyrics in Mandarin became an unexpected vehicle for language development, distinct from formal classroom instruction. A 21-year-old female participant from Thailand, a regular choir member, explained:

“*The Chinese classes were so hard, the memorisation, so dull. However, when learning Chinese choir songs, I find that I remember the words because they are connected to the melody and emotion. Singing trained my ear to tones, which greatly improved my pronunciation. Now, when I speak Chinese, there is better comprehension, and also I am more confident to go out in the city. The words just stay in my brain differently when they have a melody.*” (P02, female, 21, Thailand, regular choir).

A 27-year-old male participant from Zimbabwe, who attended choir occasionally, described a similar experience with a specific song:

“*The very first Chinese song that I learned was ‘Jasmine Flower.’ I initially did not understand all the words, but my choir conductor explained that it was about the girl’s beauty, purity, and shyness. It felt like a gift to learn that song. It was not just a word; it was a feeling, part of Chinese culture that I could have with me. Now when I hear Chinese, I don’t just hear sounds. I hear the emotion behind the words more.*” (P09, male, 27, Zimbabwe, occasional choir).

##### 3.6.4.2 Subtheme 3.2: understanding cultural values

Participants described how engagement with Chinese vocal repertoire provided insight into cultural values, aesthetics, and social norms that helped demystify aspects of Chinese society. A 28-year-old male participant from Morocco articulated this with reference to Chinese choral traditions:

“*We sang folk songs from different regions. I realised ‘this reflects something beautiful in their culture’ rather than thinking it’s weird or not understanding why people behave the way they do. The music helped me understand the Chinese emphasis on harmony, on fitting into the group. Before, I was frustrated when people didn’t express their opinions directly. But when you sing a piece that requires everyone to blend, you understand why that matters.*” (P16, male, 28, Morocco, regular choir).

A 26-year-old female participant from Mali, who attended choir frequently, extended this observation to the contrast between Chinese and Western musical aesthetics:

“*Chinese choir pieces focus on blending, not the soloist. It taught me why group harmony is valued and made me less frustrated when things did not go my way. In my home country, we value individual expression you want to stand out. Here, the music is beautiful when everyone comes together, when no one stands out. That taught me something about Chinese society that no textbook could. It helped me understand why people interact the way they do.*” (P17, female, 26, Mali, frequent choir).

##### 3.6.4.3 Subtheme 3.3: cultural hybridity and meaning-making

A smaller but analytically significant group of participants (seven of 22) described actively integrating their own cultural backgrounds with Chinese musical elements, creating hybrid forms that enabled identity negotiation. A 26-year-old female participant from India, who studied traditional voice, described this process:

“*My teacher encouraged me to bring my own tradition. We worked on Indian ragas and then found Chinese songs that used similar scales. I’m creating work that belongs to both cultures, giving my time in China meaning beyond a degree. It’s not about choosing one culture or the other. It’s about finding what emerges between them. That feels like real integration, not just survival.*” (P04, female, 26, India, traditional voice lessons).

A 32-year-old male participant from Ghana, a regular choir member, described a particularly meaningful collaborative project:

“*Our choir fused a Chinese tune with African beats for a cultural festival. It showed we could honour both traditions and figure out how to integrate our cultures. The Chinese students in the choir were excited they had never heard their folk songs with Ghanaian drumming. And we international students felt like we were contributing something, not just adapting. It was the first time I felt like my culture was seen as valuable here, not just something I brought with me that I needed to put aside.*” (P20, male, 32, Ghana, regular choir).

This subtheme suggests that for some participants, vocal education enabled a form of bicultural competence that goes beyond stress reduction to encompass positive identity development and creative agency.

#### 3.6.5 Theme interplay and contradictions

Across the three themes, several patterns of interaction and tension emerged. First, Emotional Expression and Release and Social Connection and Belonging were mutually reinforcing for most participants: the emotional vulnerability of singing with others often deepened social bonds, while the safety of those bonds enabled more profound emotional expression. As one participant noted, “*I wouldn’t have been able to cry in rehearsal if I didn’t trust the people around me. And the fact that they accepted my tears made me trust them more*” (P03, female, 22, Algeria). However, for participants who did not develop this trust such as P21, the Kenyan participant who attended only three rehearsals the potential for emotional expression remained unrealised, and the social context of group singing became a source of additional distress rather than support.

Second, Cultural Bridging Through Music intersected with both emotional and social dimensions. Several participants described how learning Chinese songs enabled them to connect emotionally with host-country peers (“*When we sing a Chinese folk song together, I feel like I understand a little of what they feel. It’s not just words; it’s a shared emotional experience*” P02, female, 21, Thailand) and to navigate social interactions with greater confidence (“*Knowing the words to popular Chinese songs became a way to connect with classmates outside the choir. It was like having a key to a door I couldn’t open before*” P09, male, 27, Zimbabwe). Third, a notable contradiction emerged regarding the function of Chinese repertoire. While most participants described engagement with Chinese music as facilitating cultural integration, two participants reported discomfort with repertoire that they perceived as politically or ideologically charged. A 30-year-old female participant from the United States, who had discontinued choir participation, noted:

“*Some of the songs we sang felt like propaganda. I didn’t want to be singing things I didn’t believe in. It made me uncomfortable, and eventually, I stopped going. I didn’t know how to raise it without causing offence.*” (P22, female, 30, USA, former occasional) This counter-narrative, while representing a minority perspective, is analytically significant. It suggests that the cultural bridging function of vocal education is not automatic but contingent on participants’ perception of repertoire as authentic and personally meaningful. For this participant, the perceived politicisation of repertoire became a barrier to the very integration that vocal education promised a tension that may be particularly salient for students from contexts with differing political perspectives on China.

#### 3.6.6 Integration of qualitative and quantitative findings

The qualitative themes elaborated above provide experiential depth to the quantitative mediation findings. As presented in Table 3 (Joint Display), Emotional Expression and Release corresponds to the quantitative pathway through emotion regulation, illustrating how singing facilitates both immediate catharsis and sustained emotional skill development. Social Connection and Belonging align with the acculturative stress pathway, showing how vocal groups provide practical and emotional resources that buffer against the stressors of cultural transition. Cultural Bridging Through Music represents an emergent dimension not fully captured by the quantitative mediation model, suggesting that vocal education may also promote wellbeing through positive identity development and cultural meaning-making a pathway warranting further investigation in future longitudinal research.

**Table 3:** Integration of quantitative and qualitative findings.

Quantitative finding (path)	Quantitative effect	Qualitative theme (mechanism)	Qualitative evidence summary	Meta-inference (integration)
Vocal Ed → emotion regulation	*β* = 0.45***	Emotional expression & release	Singing provides cathartic release and structured emotional processing; helps identify and name feelings.	Vocal education participation is associated with greater emotional awareness and regulatory skills through both physiological release and cognitive reflection on emotional content in music.
Vocal Ed → acculturative stress	*β* = −0.40***	Social connection & belonging	Choir creates shared identity transcending cultural boundaries; provides practical support networks.	Group singing fosters inclusive communities that buffer against isolation and provide tangible resources, directly reducing acculturative stress.
Emotion regulation → wellbeing	*β* = 0.37***	Emotional expression & release	Participants transfer skills from singing (identifying emotions, physical release) to daily coping.	Enhanced regulatory capacities enable students to manage cross-cultural challenges more effectively, improving daily psychological functioning.
Acculturative stress → wellbeing	*β* = −0.33***	Cultural bridging through music	Understanding Chinese music and values reduces frustration; hybrid performances create meaning.	Cultural learning through song (identified qualitatively) may transform potential stressors into opportunities for appreciation and identity integration; this represents an emergent theme warranting quantitative validation in future research.
Vocal Ed → wellbeing (direct)	*β* = 0.15**	All themes	Holistic experience combining emotional, social, and cultural benefits.	Beyond mediated pathways, vocal participation offers intrinsic enjoyment and meaning that directly enhances wellbeing.

## 4 Discussion

This study examined whether participation in vocal education is associated with psychological wellbeing among international students in China and investigated the mediating roles of emotion regulation and acculturative stress. Using a sequential explanatory mixed-methods design, the findings demonstrate that vocal education participation is positively associated with psychological wellbeing, and that this relationship is statistically mediated by emotion regulation and reduced acculturative stress. Qualitative findings further elucidate these pathways, revealing mechanisms of emotional expression and release, social connection and belonging, and cultural bridging through music. Together, the quantitative and qualitative strands converge to provide a theoretically grounded account of how arts-based engagement may support mental health during cultural transition.

### 4.1 Theoretical integration: emotion regulation as a mechanism of change

The finding that emotion regulation significantly mediated the association between vocal education participation and psychological wellbeing aligns with and extends (Gross, 2015), the extended process model of emotion regulation. Within this framework, adaptive emotion regulation involves a sequence of identification, selection, and implementation of strategies to modulate emotional responses. Vocal education, as described by participants in the qualitative strand, appears to engage multiple stages of this process. First, the act of singing particularly in formal or semi-formal settings requires sustained attentional focus on emotional content embedded in lyrics and musical structure, thereby facilitating *situation selection* and *attentional deployment*. Second, participants described how vocal training encouraged them to identify, label, and articulate nuanced emotional states (e.g., distinguishing loneliness from frustration), which corresponds to the *cognitive change* stage of reappraisal. Third, the physiological release reported during and after singing often described as “feeling lighter” or “letting go” reflects *response modulation*, the final stage in Gross’s model. Importantly, participants reported that these regulatory skills transferred beyond the musical context into daily life, suggesting that vocal education may serve as a scaffold for developing generalised emotion regulation capacities. This finding resonates with previous work indicating that structured musical engagement can foster emotional intelligence and regulatory flexibility (Perkins et al., 2020; Soufi Amlashi and Forstmeier, 2025), but it extends that literature by situating these processes within the specific acculturative challenges faced by international students.

### 4.2 Acknowledging directional ambiguity

Before interpreting the mediation findings, it is essential to acknowledge an alternative explanation that the cross-sectional data cannot rule out: students with higher baseline emotion regulation capacity and lower acculturative stress may be more likely to join and remain in vocal education programs. Emotionally regulated individuals may feel more comfortable in group singing contexts, and those experiencing less acculturative stress may have more cognitive and emotional resources to devote to extracurricular activities. This selection effect could be consistent with the pattern of associations observed here, such that vocal education participation is associated with wellbeing even if pre-existing differences underlie the relationship. The alternative model testing (Model A: reverse causation) provided poorer fit to the data, which somewhat mitigates this concern, but fit comparisons do not establish causality. Longitudinal studies tracking international students before and after vocal education participation are needed to examine whether the observed associations reflect selection effects or other directional processes.

### 4.3 Theoretical integration: belonging and acculturative stress

The mediating role of acculturative stress and, by extension, the qualitative theme of social connection and belonging is theoretically grounded in Berry’s (1997) acculturation framework and the social identity approach to health (Jetten et al., 2017). According to Berry’s model, acculturative stress arises when the demands of cultural transition exceed an individual’s resources for coping. In the present study, vocal education participation appeared to enhance both social and psychological resources that buffer against such stress. Quantitatively, participation was negatively associated with acculturative stress; qualitatively, participants described how choir and vocal groups provided tangible social support (e.g., assistance with banking, visa procedures), a sense of shared identity that transcended national boundaries, and a reliable source of positive intergroup contact with host-country peers. These experiences align closely with the *social identity approach*, which posits that group memberships confer psychological resources such as belonging, meaning, and collective self-esteem that are critical for mental health (Jetten et al., 2017) For international students, who often experience dislocation from pre-existing support networks, the formation of a new, meaningful social identity within a vocal group may be particularly consequential. Moreover, the finding that acculturative stress mediated the vocal education wellbeing relationship suggests that the statistically associated benefits of group singing operate not only through general social support but also through the specific alleviation of culture-related stressors. This is consistent with previous research showing that positive intergroup contact and peer support are key determinants of cross-cultural adaptation (Zhang and Goodson, 2011).

### 4.4 Integrating emotion regulation and acculturative stress: toward a sequential model

While the primary analysis tested a parallel mediation model, the exploratory sequential mediation analysis suggested that emotion regulation and acculturative stress may operate in sequence: vocal education participation was associated with improved emotion regulation, which in turn was associated with reduced acculturative stress, and ultimately with greater psychological wellbeing. Although cross-sectional data preclude causal inference, this sequential pathway is theoretically compelling. It implies that the regulatory skills cultivated through vocal education may enable students to appraise and respond to acculturative challenges more effectively, thereby reducing the cumulative stress of cultural transition. This interpretation is consistent with research demonstrating that emotion regulation skills predict acculturative adjustment (Cheung et al., 2020), and that deficits in emotion regulation are associated with higher acculturative stress (Soufi Amlashi and Forstmeier, 2025). The qualitative narratives reinforced this interconnected view: participants described how learning to name and manage emotions through singing helped them approach cross-cultural difficulties (e.g., language barriers, unfamiliar social norms) with greater composure and problem-solving orientation, rather than with overwhelming distress. Thus, rather than operating as entirely independent mechanisms, emotion regulation and acculturative stress may represent linked processes in a dynamic system where enhanced regulatory capacity reduces the perceived and actual burden of cultural adaptation.

### 4.5 Cultural bridging as an emerging qualitative dimension

It is important to emphasize that this mechanism emerged from the qualitative analysis only; the quantitative mediation model did not include a direct measure of cultural bridging. Future research should develop quantitative measures of cultural bridging through music to test whether this pathway operates independently of or in conjunction with emotion regulation and acculturative stress. Participants described how engagement with Chinese vocal repertoire facilitated language acquisition, provided insights into cultural values (e.g., the emphasis on group harmony over individual expression), and enabled the creation of hybrid cultural forms that integrated their heritage with host-country traditions. These processes resonate with the *cultural learning* and *creative adaptation* dimensions of acculturation theory (Sam and Berry, 2006), which emphasise that successful adaptation involves not merely stress reduction but also active meaning-making and identity negotiation. For international students, vocal education thus appears to offer a culturally embedded context for experiential learning one that transforms potentially disorienting cultural encounters into opportunities for personal growth and integration. This finding suggests that future theoretical models of acculturation and wellbeing among sojourners should incorporate arts-based activities as sites of both stress buffering and positive identity development.

### 4.6 Implications for theory and practice

Theoretically, the study advances understanding of how arts engagement promotes psychological wellbeing by specifying two interconnected mediating pathways emotion regulation and acculturative stress and by grounding them in established frameworks of emotional processing, social identity, and acculturation. In doing so, it responds to calls in the literature for greater theoretical precision regarding the mechanisms linking participatory arts to mental health outcomes (Clift et al., 2016; Perkins et al., 2020). Practically, the findings suggest that university-based vocal education programs may serve as accessible, culturally resonant interventions to support the mental health of international students. Such programs may be particularly valuable in contexts where traditional counselling services are underutilised due to stigma or cultural barriers. To maximise impact, vocal programs should be designed to foster both emotional expression (e.g., through structured processing of song content) and inclusive social belonging (e.g., by intentionally integrating international and domestic students, and by including repertoire from diverse cultural traditions). The qualitative finding that participants valued both emotional and intercultural dimensions of the experience suggests that programs attending to these dual aspects are likely to yield the greatest psychological benefits.

## 5 Study limitations

Several limitations mitigate the interpretation of these findings. First, the cross-sectional design does not allow for causal inference or temporal precedence among variables. Although alternative model testing indicated that the proposed directional sequence fit the data better than the reverse causal model, longitudinal or experimental designs are needed to establish directional relations. Second, comparisons across participation groups indicated that regular participants reported higher Chinese language proficiency than non-participants. Although this variable was included as a covariate in the primary analyses, the possibility of residual confounding cannot be excluded. Higher levels of linguistic confidence may be associated with both greater participation in vocal activities and more favorable wellbeing scores, warranting cautious interpretation of these associations. Third, self-report measures, regardless of their reliability and validity, are vulnerable to response bias. Fourth, the composite measure of vocal education participation allows capture of multiple dimensions of engagement but may mask distinctions between modes, such as solo versus group or formal versus informal participation. Fifth, while the sample was diverse, it is limited to respondents from three universities in a single Chinese city and may not generalise to international students in other settings or host countries.

## 6 Conclusion

This mixed-method study provides evidence that involvement in vocal education is positively associated with international students’ psychological health, with emotion regulation and acculturative stress statistically mediating this association. These findings show that emotional expression and peer communication mediate the link between vocal education and wellbeing: singing strengthens emotion regulation (adaptive reappraisal of acculturative challenges) and group singing reduces acculturative stress via social bonding and collective identity illustrating how structured musical engagement translates expressive and communicative processes into mental health benefits. Cross-sectional design precludes causal interpretation of these mediation pathways. Perhaps more than artistic enrichment, vocal programs are associated with psychological resources that may reflect or facilitate adaptation to new cultural environments. Qualitative findings revealed emotional release, social connection, and cultural bridging as participant experiences that explained the quantitative evidence of mediation pathways. The study has practical implications for universities looking for accessible, culturally tailored strategies to support the mental health of international students. Arts-based activities, such as vocal education, can be a valuable adjunct to traditional mental health services, especially given the growing global population of international students. Despite the limitations of causal inference stemming from its cross-sectional design, this study provides novel evidence that cultivating opportunities for vocal education may be an effective pathway to enhancing international students’ wellbeing in global higher education.

## Data Availability

The original contributions presented in the study are included in the article/supplementary material, further inquiries can be directed to the corresponding author.

## References

[ref1] Antoninis M. Alcott B. Al Hadheri S. April D. Fouad Barakat B. Barrios Rivera M. (2023) Global Education Monitoring Report 2023: Technology in education: A tool on whose terms? Available online at: https://discovery.ucl.ac.uk/id/eprint/10195257/

[ref2] Awasthi P. Mukherjee M. Srivastava N. Saxena S. (2022). Predictive role of emotion-regulation in acculturative stress and spiritual well-being of international students. PURUSHARTHA-A J. Manag. Ethics Spirituality 15, 126–138. doi: 10.21844/16202115207

[ref3] Berry J. W. (1997). Immigration, acculturation, and adaptation. Appl. Psychol. 46, 5–34. doi: 10.1111/j.1464-0597.1997.tb01087.x

[ref4] Braun V. Clarke V. (2021). Thematic Analysis: A Practical Guide. American Psychological Association.

[ref5] Cheung R. Y. Bhowmik M. K. Hue M.-T. (2020). Why does acculturative stress elevate depressive symptoms? A longitudinal study with emotion regulation as a mediator. J. Couns. Psychol. 67, 645–652. doi: 10.1037/cou0000412, 31855019

[ref6] Clift S. Hancox G. (2010). The significance of choral singing for sustaining psychological wellbeing: findings from a survey of choristers in England, Australia and Germany. Music Perform. Res. 3, 93–100. doi: 10.1016/j.puhe.2016.03.022

[ref7] Clift S. Page S. Daykin N. Peasgood E. (2016). The perceived effects of singing on the health and well-being of wives and partners of members of the British armed forces: a cross-sectional survey. Public Health 138, 93–100. doi: 10.1016/j.puhe.2016.03.022, 27137872

[ref8] Creswell J. W. Clark V. P. (2007). Mixed Methods Research. Thousand Oaks, CA: cambridgeenglish.org.

[ref9] Diamantopoulos A. Siguaw J. A. (2006). Formative versus reflective indicators in organizational measure development: a comparison and empirical illustration. Br. J. Manage. 17, 263–282. doi: 10.1111/j.1467-8551.2006.00500.x

[ref10] Dingle G. A. Williams E. Jetten J. Welch J. (2017). Choir singing and creative writing enhance emotion regulation in adults with chronic mental health conditions. Br. J. Clin. Psychol. 56, 443–457. doi: 10.1111/bjc.12149, 28722166

[ref1101] Enders C. K. (2010). Applied Missing Data Analysis. New York: The Guilford Press. Available online at: https://www.guilford.com/books/Applied-Missing-Data-Analysis/Craig-Enders/9781462549863

[ref11] Fancourt D. Ockelford A. Belai A. (2014). The psychoneuroimmunological effects of music: a systematic review and a new model. Brain Behav. Immun. 36, 15–26. doi: 10.1016/j.bbi.2013.10.014 24157429

[ref12] Fryling D. S. (2015). Persistence in Choral Music: An Investigation into Psychological and Sociological Factors Involved in Choral Membership. Hempstead, New York: Hofstra University.

[ref13] Fung S.-f. Kong C. Y. W. Liu Y.-m. Huang Q. Xiong Z. Jiang Z. . (2022). Validity and psychometric evaluation of the Chinese version of the 5-item WHO well-being index. Front. Public Health 10:872436. doi: 10.3389/fpubh.2022.872436, 35433612 PMC9005828

[ref14] Gao H. De Wit H. (2017). China and international student mobility. Int. High. Educ. 90, 3–5. doi: 10.6017/ihe.2017.90.9992

[ref15] Gratz K. L. Roemer L. (2004). Multidimensional assessment of emotion regulation and dysregulation: development, factor structure, and initial validation of the difficulties in emotion regulation scale. J. Psychopathol. Behav. Assess. 26, 41–54. doi: 10.1023/b:joba.0000007455.08539.94

[ref16] Gross J. J. (2015). Emotion regulation: current status and future prospects. Psychol. Inq. 26, 1–26. doi: 10.1080/1047840X.2014.940781

[ref17] Guest G. Bunce A. Johnson L. (2006). How many interviews are enough? An experiment with data saturation and variability. Field Methods 18, 59–82. doi: 10.1177/1525822X052799

[ref18] Han R. Bos V. Wiebusch F. Broughton M. C. Dingle G. A. (2025). The impact of choir singing on international students’ sense of belonging, loneliness, and wellbeing: a controlled evaluation of UQ voices. Behav. Sci. 15:575. doi: 10.3390/bs15050575, 40426352 PMC12108629

[ref19] Hang Y. Zhang X. (2023). Intercultural competence developmental processes of university and college students as three types of transition–a systematic review. Int. J. Intercult. Relat. 92:101748. doi: 10.1016/j.ijintrel.2022.101748

[ref20] Jackson D. L. (2003). Revisiting sample size and number of parameter estimates: some support for the N: q hypothesis. Struct. Equ. Model. Multidiscip. J. 10, 128–141. doi: 10.1207/S15328007SEM1001_6

[ref21] Jetten J. Haslam S. A. Cruwys T. Greenaway K. H. Haslam C. Steffens N. K. (2017). Advancing the social identity approach to health and well-being: progressing the social cure research agenda. Eur. J. Soc. Psychol. 47, 789–802. doi: 10.1002/ejsp.2333

[ref22] Jiang Q. Li Y. Shypenka V. (2018). Loneliness, individualism, and smartphone addiction among international students in China. Cyberpsychol. Behav. Soc. Netw. 21, 711–718. doi: 10.1089/cyber.2018.011, 30328694

[ref23] Kreutz G. (2014). Does singing facilitate social bonding? Music Med. 6, 51–60. doi: 10.47513/mmd.v6i2.180

[ref24] Kreutz G. Bongard S. Rohrmann S. Hodapp V. Grebe D. (2004). Effects of choir singing or listening on secretory immunoglobulin a, cortisol, and emotional state. J. Behav. Med. 27, 623–635. doi: 10.1007/s10865-004-0006-9, 15669447

[ref25] MacRitchie J. Breaden M. Milne A. J. McIntyre S. (2020). Cognitive, motor and social factors of music instrument training programs for older adults’ improved wellbeing. Front. Psychol. 10:2868. doi: 10.3389/fpsyg.2019.02868, 31998175 PMC6968490

[ref26] Pearce E. Launay J. Dunbar R. I. (2015). The ice-breaker effect: singing mediates fast social bonding. R. Soc. Open Sci. 2, 1–9. doi: 10.1098/rsos.150221, 26587241 PMC4632513

[ref27] Perkins R. Mason-Bertrand A. Fancourt D. Baxter L. Williamon A. (2020). How participatory music engagement supports mental well-being: a meta-ethnography. Qual. Health Res. 30, 1924–1940. doi: 10.1177/1049732320944, 32755294 PMC7502980

[ref28] Podsakoff P. M. MacKenzie S. B. Podsakoff N. P. (2012). Sources of method bias in social science research and recommendations on how to control it. Annu. Rev. Psychol. 63, 539–569. doi: 10.1146/annurev-psych-120710-100452, 21838546

[ref29] Sam D. L. Berry J. W. (2006). The Cambridge Handbook of Acculturation Psychology. Cambridge University Press.

[ref30] Sandhu D. S. Asrabadi B. R. (1994). Development of an acculturative stress scale for international students: preliminary findings. Psychol. Rep. 75, 435–448. doi: 10.2466/pr0.1994.75.1.435, 7809315

[ref31] Satorra A. Saris W. E. (1985). Power of the likelihood ratio test in covariance structure analysis. Psychometrika 50, 83–90. doi: 10.1007/BF02294150

[ref32] Smith R. A. Khawaja N. G. (2011). A review of the acculturation experiences of international students. Int. J. Intercult. Relat. 35, 699–713. doi: 10.1016/j.ijintrel.2011.08.004

[ref33] Song L. Peng C. Hai Y. Wang J. Zhao J. (2025). The influence of perceived social support on anxiety among international students: the mediating role of communicative adaptability. Front. Psychol. 16:1498261. doi: 10.3389/fpsyg.2025.1498261, 41602731 PMC12833321

[ref34] Soufi Amlashi R. Forstmeier S. (2025). The relationship of acculturative stress with meaning in life through the mediating role of difficulties in emotion regulation and meaning-centered coping style among international students in Germany. Front. Psychol. 16:1639194. doi: 10.3389/fpsyg.2025.1639194, 40823399 PMC12354631

[ref35] Sullivan C. Kashubeck-West S. (2015). The interplay of international students’ acculturative stress, social support, and acculturation modes. J. Int. Stud. 5, 1–11. doi: 10.32674/jis.v5i1.438

[ref36] Suyuhan W. Khoso A. R. Jintu G. Bhutto S. (2026). The mental health crisis in global higher education: understanding and mitigating academic load stress among international students from Asia and Africa in Nanjing China. Front. Psychol. 17:1707944. doi: 10.3389/fpsyg.2026.1707944, 41658377 PMC12873712

[ref37] Tehseen S. Ramayah T. Sajilan S. (2017). Testing and controlling for common method variance: a review of available methods. J. Manage. Sci. 4, 142–168. doi: 10.20547/jms.2014.1704202

[ref38] Topp C. W. Østergaard S. D. Søndergaard S. Bech P. (2015). The WHO-5 well-being index: a systematic review of the literature. Psychother. Psychosom. 84, 167–176. doi: 10.1159/000376585, 25831962

[ref39] Vasiou A. Vasilaki E. Mastrothanasis K. Gkontelos A. (2025). Behind university students’ academic success: exploring the role of emotional intelligence and cognitive test anxiety. Trends Higher Educ 4:56. doi: 10.3390/higheredu4030056

[ref40] Williams E. Dingle G. A. Clift S. (2018). A systematic review of mental health and wellbeing outcomes of group singing for adults with a mental health condition. Eur. J. Pub. Health 28, 1035–1042. doi: 10.1093/eurpub/cky115, 29982515

[ref41] Yao Z. (2024). *Promoting the Emotional Expression of Primary School Learners in Singing Activity* (doctoral dissertation), Vytautas Magnus University].

[ref42] Yu X. Ishiwata T. Zhang X. Zhang D. Xu L. Su Y. . (2025). ‘More than just music’: a multimodal intervention supporting adolescents with autism spectrum disorder in a community-based program in Shanghai. Int. J. Dev. Disabilities 1–16, 1–16. doi: 10.1080/20473869.2025.2561160

[ref43] Zhang J. Goodson P. (2011). Predictors of international students’ psychosocial adjustment to life in the United States: a systematic review. Int. J. Intercult. Relat. 35, 139–162. doi: 10.1016/j.ijintrel.2010.11.011

